# Psychosocial outcomes of group-based physical activity among individuals with substance use disorders: a scoping review

**DOI:** 10.1186/s13722-026-00685-z

**Published:** 2026-06-16

**Authors:** Eirin Bryn, Marco Tagliabue, Børge Strømgren

**Affiliations:** Department of Behavioural Sciences, Faculty of Health Sciences, OsloMet - Oslo Metropolitan University, PO box 4 St. Olavs plass, Oslo, 0130 Norway

**Keywords:** Group-based physical activity, Psychosocial factors, SUDs, Alternative reinforcers, Motivating operations, Social support, Cohesion

## Abstract

Substance use disorders (SUDs) are increasing globally, and social support as well as access to substance-free activities are essential for sustained recovery. Mental health services should therefore be effective, accessible, and supportive of psychosocial functioning. The aim of this scoping review was to map the extent of research examining psychosocial outcomes associated with group-based physical activity among individuals with SUDs and to identify psychosocial factors reported to enhance the reinforcing value of such activities. In addition, the review assessed the extent to which included studies emphasized interaction, communication, cooperation, and group cohesion, and whether interventions were delivered alongside other psychosocial treatments. Following PRISMA-ScR guidelines, searches were conducted in MEDLINE, PsycINFO, Scopus, and the Cochrane Library for publications from 2010 to 2025. Eighteen studies met the inclusion criteria. Across studies, psychosocial factors such as structured and inclusive environments, professional guidance, perceived autonomy, and social support were commonly reported as contributing to engagement and positive psychosocial outcomes associated with group-based physical activity. However, relatively few studies explicitly emphasized group-level processes, and most interventions were delivered in combination with other psychosocial treatments, limiting the ability to isolate group-specific effects. This review highlights a knowledge gap concerning the social components of group-based physical activity interventions and their role in shaping psychosocial outcomes among individuals with SUDs.

**Supplementary Information:**

The online version contains supplementary material available at 10.1186/s13722-026-00685-z.

## Introduction

Substance use disorders (SUDs) are an umbrella term for a chronic mental health condition encompassing both harmful use of substances and substance dependence. A diagnosis of substance dependence can be made when an individual loses control over their intake of substances, develops tolerance, and continues using those substances despite their adverse consequences [1]. Individuals with SUDs frequently experience comorbid mental and physical health problems, leading to significant consequences for themselves, their families, and society. The associated costs for somatic and mental health services at both the municipal and specialist levels are substantial [1]. The number of individuals with SUDs is increasing globally [2], and a recent international meta-analysis indicates that contemporary psychosocial treatments for SUDs are characterized by high dropout rates and elevated relapse rates [3].

SUDs comprise a chronic class of mental health conditions characterized by harmful substance use and substance dependence, with the latter involving loss of control over intake, tolerance development, and continued use despite adverse consequences [1]. Individuals with SUDs frequently experience comorbid mental and physical health problems, resulting in substantial personal, familial, and societal burden. The associated costs for municipal and specialist health services are considerable [1], and the global prevalence of SUDs continues to rise [2]. Despite the availability of established psychosocial treatments, dropout and relapse rates remain high [3], highlighting the need for complementary approaches that may enhance engagement, retention, and psychosocial functioning.

Health and well-being are shaped by contextual factors, including social, environmental, and economic conditions, and are foundational for individual functioning and societal participation (United Nations Association of Norway, [4]). In Norway, national mental health policy emphasizes accessible, effective services with an explicit focus on improving mental well-being and quality of life [5]. This emphasis aligns with international policy frameworks, including the *Comprehensive Mental Health Action Plan 2013–2030* published by the World Health Organization [6], which highlights community-based, recovery-oriented services that promote social inclusion and well-being, and the United Nations Office on Drugs and Crime’s recovery framework, which underscores the role of social connectedness, meaningful activity, and participation in sustained recovery from substance use disorders [7]. Within this broader international context, interventions that promote social inclusion, emotional well-being, and sustainable behavioral engagement are particularly relevant for individuals with SUDs.

Psychosocial impairment is common among individuals with SUDs, including elevated symptoms of anxiety and depression, disrupted interpersonal relationships, and pronounced experiences of loneliness following treatment completion [8]. Social environments that facilitate positive interaction and support have been shown to strengthen adaptive behavioral patterns and improve mental health outcomes [9]. Perceived social support from family members, peers, and communities is associated with reduced relapse risk and may contribute to the maintenance of substance-free behavior [10]. Accordingly, increasing access to substance-free activities that support emotional well-being, physical health, and positive social relationships is considered an important component of recovery-oriented care [11, 12].

Physical activity is well established as a contributor to overall physical and mental health [13]. When integrated into substance use treatment contexts, physical activity has been associated with reductions in cravings, improvements in mood regulation, and decreases in symptoms of anxiety and depression [14–16]. Importantly, the psychosocial benefits of physical activity appear to be influenced by how activities are structured and experienced. Activities perceived as enjoyable, socially supportive, and meaningfully embedded in everyday life are more likely to promote sustained engagement and psychological benefit [17].

Group-based physical activity has been associated with improvements in quality of life across both clinical and non-clinical populations, whereas comparable benefits are less consistently observed in individually delivered or home-based exercise [18]. Group formats may facilitate social interaction, cooperation, communication, and shared goal-directed activity, thereby fostering group cohesion, social connectedness, and a sense of belonging [19]. From a behavioral perspective, such contexts may increase access to alternative, non-substance-related sources of reinforcement and support the development of adaptive behavioral repertoires in recovery. However, empirical investigations of group-based physical activity in SUD populations vary widely in design, outcomes measured, and theoretical emphasis.

Psychosocial factors are understood to include psychological risk factors (e.g., depressed mood, emotional distress), psychological resources (e.g., perceived mastery, self-esteem), and socially embedded resources such as social support and networks. They have been conceptualized in diverse ways across disciplines [20, 21]. Although these factors are widely recognized as relevant to recovery and well-being, the extent to which group-based physical activity interventions explicitly target or influence psychosocial outcomes among individuals with SUDs has not been systematically mapped.

To date, no review has synthesized the literature specifically examining group-based physical activity in relation to psychosocial outcomes among individuals with SUDs. The existing body of research is heterogeneous, encompassing randomized controlled trials, quasi-experimental studies, and qualitative investigations that variously emphasize outcomes, contextual factors, and intervention processes. Given this heterogeneity and the emerging nature of the field, a scoping review is a suitable approach to describe the extent, characteristics, and focus of the available evidence, without restricting inclusion to a narrow set of intervention types or outcome measures.

### Objectives

Based on the current state of the literature, the primary objectives of this scoping review were (1a) to map the extent of research examining psychosocial outcomes associated with group-based physical activity among individuals with SUDs and (1b) to identify psychosocial factors that may contribute to engagement in or perceived value of group-based physical activity. The secondary objectives were (2a) to assess the degree to which included interventions emphasized interaction, communication, cooperation, and group cohesion, and (2b) to examine whether group-based physical activity interventions were delivered in conjunction with other psychosocial treatments.

## Method

This scoping review was conducted in accordance with the *Preferred Reporting Items for Systematic Reviews and Meta-Analyses Extension for Scoping Reviews (PRISMA*-ScR). A checklist outlining all the elements reported in scoping reviews [22] is included in the related files section. Scoping reviews employ systematic search procedures similar to those used in systematic reviews but do not require risk-of-bias assessment and are not restricted to specific outcomes or intervention types [23]. The purpose of a scoping review is to identify and map the breadth of available evidence related to a particular field, topic, or research question across one or more defined contexts [24].

### Eligibility criteria

To be included, studies were required to examine the effects of group-based physical activity on psychosocial variables, such as depression, quality of life, and the experience of social support and belonging, among individuals with SUDs. Only peer-reviewed articles published in English or Norwegian between 2010 and 2025 were eligible to ensure alignment with the current international research landscape. Both quantitative and qualitative studies were included to provide a comprehensive understanding of the phenomenon.

### Population

Eligible participants were adults (≥ 18 years), regardless of gender, with SUDs, with or without comorbid mental disorders. Participants could be engaged in inpatient or outpatient treatment or not receiving treatment at the time of the study. Individuals could be currently using substances or receiving concurrent medication-assisted treatment (e.g., methadone or buprenorphine), with or without the use of additional substances. Substances included both legal and illegal psychoactive drugs; nicotine and caffeine were excluded due to their comparatively limited psychoactive effects.

### Intervention

Eligible interventions involved group-based physical activity, a necessary criterion for assessing social factors. Studies focusing exclusively on biological or physiological outcomes, cravings, or substance use, as well as studies combining physical activity with other interventions such as dietary changes or pharmacological treatments, were excluded. Studies involving populations other than individuals with SUDs were also excluded to ensure that the findings remained specific and relevant to the target group.

### Search strategy

To identify relevant studies, searches were conducted between January 2, 2025, and the final search on January 21, 2025. Four databases were searched: MEDLINE (Ovid), PsycINFO (Ovid), Scopus, and the Cochrane Library (see Appendix A).

An initial search was conducted in the International Prospective Register of Systematic Reviews (PROSPERO) and the clinical knowledge resource *UpToDate*. The search terms “substance use” OR “alcohol use” OR “drug addiction” AND “group training” OR “group exercise” were used. No relevant reviews addressing SUDs and group-based physical activity were identified.

Keywords were then selected based on previous reviews of similar interventions involving group-based physical activity among individuals with SUDs. These included English synonyms and related terms referring to both SUDs and group-based physical activity. Searches combining broad physical activity terms (e.g., exercise, physical activity) with substance-related terms (e.g., SUDs, alcohol use disorder) generated a very large number of records. In contrast, searches using more specific terms related to group-based activities (e.g., *group exercise*, *soccer*) combined with substance-related terms yielded very few records.

During the search process, both subject headings (e.g., MeSH terms) and keywords were used in the Cochrane Library and PsycINFO (Ovid). In Scopus and MEDLINE (Ovid), only keywords were used. In MEDLINE (Ovid), the use of subject headings related to group-based activity produced irrelevant results, and therefore only keywords were applied. Truncation was used where appropriate to ensure a comprehensive search. The final search conducted on January 21, 2025, combined various subject headings and keywords (see Appendix B).

### Study selection

The final set of search results was imported into EndNote (Version 21), where duplicates were first removed automatically by a librarian and subsequently checked manually. Study selection proceeded in three stages.

At the first stage, the first author screened titles and abstracts to remove records that clearly did not meet the predefined eligibility criteria. This included studies that did not involve group-based physical activity, did not target individuals with SUDs, or did not assess psychosocial variables. Study protocols and systematic reviews were also excluded at this stage.

At the second stage, a fellow student and the first author independently screened titles and abstracts of the remaining articles using the eligibility criteria. Interrater reliability was assessed using Cohen’s kappa [25], a statistical measure of agreement between two independent reviewers that accounts for the possibility of chance agreement.

At the third stage, full-text articles were independently screened by both reviewers to determine whether they met the specified eligibility criteria. Any uncertainties regarding study inclusion were discussed continuously to ensure that all relevant studies were retained. Throughout the screening process, we focused on three core variables: whether the physical activity intervention was group-based, whether participants had SUDs and whether the study measured the effects of group-based physical activity on psychosocial variables.

### Data charting and extracted elements

The first author developed a data-charting table to extract and organize information relevant to the primary and secondary objectives of this scoping review. First, key characteristics of the included studies were charted, including authors, year of publication, country, research design, sample size, and participants’ treatment status (inpatient, outpatient, or not in treatment). Additional information was collected regarding the type of group-based physical activity, the methods used to measure the intervention’s effects on psychosocial variables, and the reported impact on these outcomes. The reported impacts of interventions on psychosocial variables within the included studies were synthesized and categorized. These categories included: impact on psychosocial factors, impact on social factors, impact on psychological factors, or no observed impact on social or psychological factors.

Subsequently, we extracted whether the included studies mentioned psychosocial factors that appeared to enhance the reinforcing value of group-based physical activity; whether the studies emphasized interaction, communication, cooperation, and group cohesion, whether the interventions were implemented alongside other psychosocial treatments, and if the interventions measured group processes.

## Results

### Selection process

The final database search yielded 2,894 records. Of these, 555 duplicates were automatically removed and an additional 108 were removed manually, resulting in a total of 663 duplicates. At the first screening stage, titles and abstracts of the remaining 2,231 records were reviewed, leading to the exclusion of 2,124.

At the second stage, titles and abstracts of the remaining 107 studies were independently screened by both reviewers. This process resulted in the inclusion of 17 articles and the exclusion of 84, with disagreements on 6 articles. Interrater reliability was calculated as κ = 0.80, indicating substantial agreement. All 23 articles (17 with agreement and 6 with disagreement) were then independently assessed at the full-text stage until consensus was reached.

**Fig. 1:**
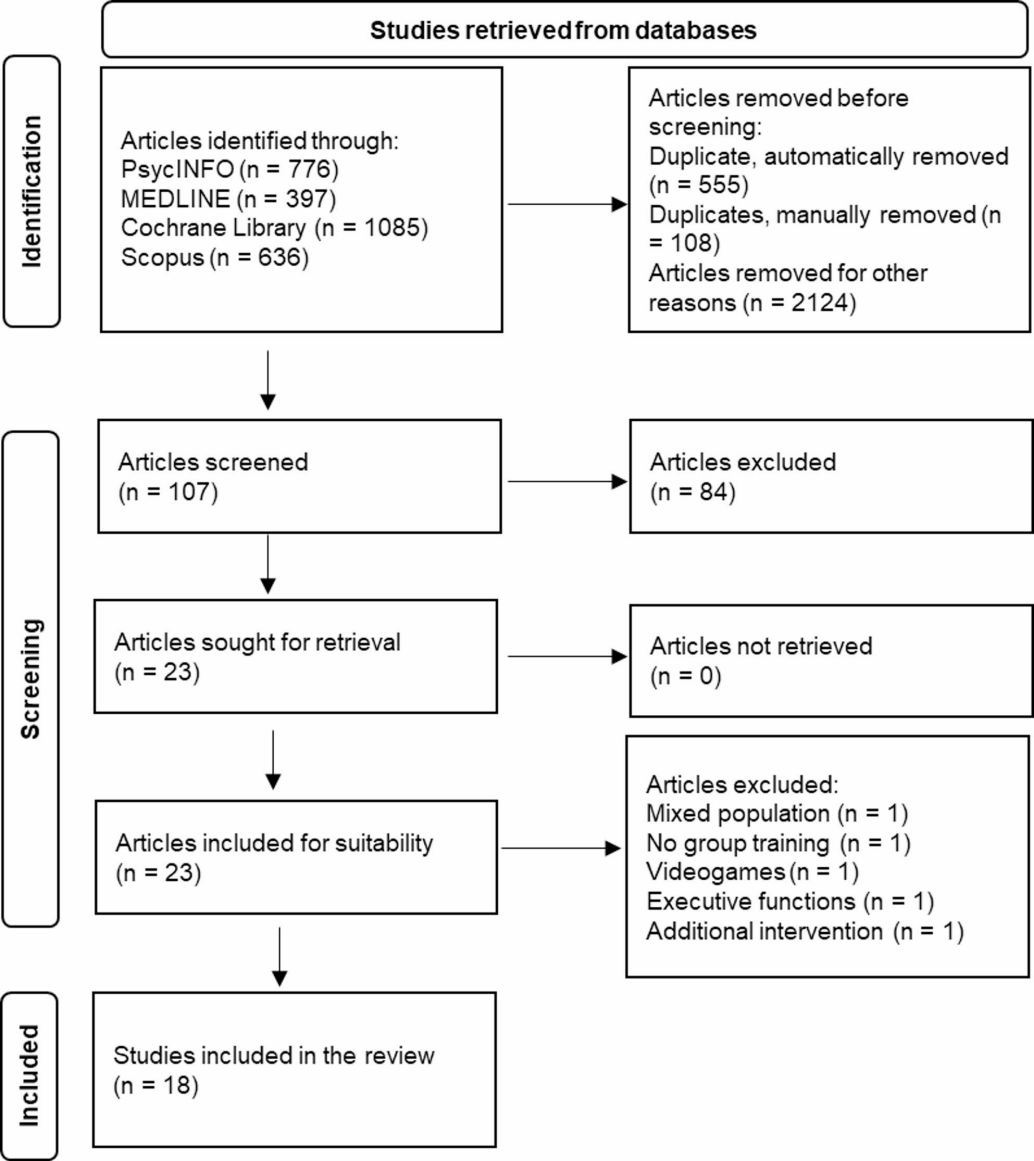
PRISMA 2020 flowchart. Note. Reproduced from "Tricco et al., 2018" convert into number

Two qualitative studies were included; in one study, participants were undergoing treatment for SUDs, and in the other, participants were either in treatment or in recovery. The final selection process resulted in 18 included studies (see Fig. 1).

### Characteristics of the included studies

Of the included studies, nine were randomized controlled trials, five were quasi-experimental, one used a mixed-methods design, and three were qualitative studies. Four studies were pilot trials. Among the studies involving participants in outpatient treatment, one was conducted in Switzerland [26] and two in Denmark [27, 28].

Twelve studies included participants in inpatient treatment, spanning across several countries and regions (please see Table 1 for detail). Among them, one study conducted in the USA [29] included participants in both inpatient and outpatient settings, as well as individuals not receiving treatment. One study from England [30] included both inpatient and outpatient participants, and one Swedish study [31] included participants not in treatment.

**Table 1:** Characteristics of the included studies and the key factors this scoping review aimed to elucidate

Author, Year	Country	Method	Sample Size	Treatment Setting	Physical Activity, Frequency, and Duration	Measurement Methods	Impact	Psychosocial Factors, Outcome	Interaction, Communication, Cooperation, and Group Cohesion	Addition to Psychosocial Treatment	Measurement of Group Processes
Colledge et al. [26]	Switzerland	RCT, pilot	*N* = 24	Outpatient	Choice between climbing, badminton, dance, and strength training, or walking and sometimes coordination games (compared to: group activities, such as board games and museum visits). 3 months, 2 × 60 min. per week	Centre for Epidemiologic Studies Depression Scale, BSCS, PSS, SF-36	No	Yes	No	Yes	No
Fitzgerald et al. [30]	England	Quasi	*N* = 43	Inpatient and Outpatient	Strength training. Average 2 months. Minimum 5 sessions	POMS-ASF, Short «grit» questionnaire	Psychososial	Yes	Yes	Yes	No
Gaihre et al. [32]	India	RCT	*N* = 96	Inpatient	Yoga (compared to running, walking, strength training, and stretching). 3 months, 6 × 90 min. per week	BSCS, HADS, FMI-14	Psychological	No	No	Yes	No
Giesen et al. [33]	Germany	Quasi	*N* = 44	Inpatient	Stationary and water cycling, strength training, coordination, and flexibility training, plus two day trips per year (compared to TAU, and an MCG without treatment and training). 12 months, 2 × 60 min. per week	SF-36	Psychological	Yes	No	Yes	No
Giménez-Meseguer et al. [34]	Spain	Mixed	*N* = 47	Inpatient	Varied training and recreational games (compared to TAU). 3 months. 3 × 60–90 min. per week	SF-36. In-depth interview	Psychosocial	Yes	Yes	Yes	In-depth interviews
Huang et al., [35]	China	RCT	*N* = 90	Inpatient	Health qi gong (compared to cardio training with music and TAU). 3 months, 4 × 60 min. per week	SF-36	Psychological	No	No	Yes	No
Kiraz & Yıldırım, [36]	Turkey	RCT	*N* = 75	Inpatient	Varied (compared to TAU). 3 weeks, Pilates 2 × 60 min. and varied team sports 3 × 60 min. per week	HAM-A, HAM-D, TMQ, MAAS, ASI	Psychological	No	No	Yes	No
Monton-Martinez et al. [37]	Spain	Quasi, pilot	*N* = 26	Inpatient	Personally guided movement, strength, and cardio training, such as boxing (compared to pre-recorded online training). 3 months, 3 × 45–60 min. per week	HRQOLDA	Quality of life	Yes	No	Yes	No
Muller and Clausen, [38]	Norway	Quasi, pilot	*N* = 35	Inpatient	Walking/running, strength and ball training. 10 weeks. 3 × 30 min. per week	WHOQOL-BREF, HSCL-25, ASI	Psychosocial	Yes	No	Yes	No
Nowakowski-Sims & Bullard, [29]	USA	Qualitative	*N* = 16	Inpatient, Outpatient and Not in treatment	Mainly CrossFit, boot-camp, yoga, and mixed martial arts for a minimum of 3 months	Individual interviews	Psychosocial	Yes	Yes	Mixed	Semi-structured interviews
Piche et al. [16]	Canada	Qualitative	*N* = 13	Inpatient	Yoga, cardio, and strength training. 5 weeks, 3 × 60 min. per week	Individual interviews	Psychosocial	Yes	No	Yes	Semi-structured interviews
Roessler, [27]	Denmark	Qualitative, pilot	*N* = 38	Outpatient	Cycling and strength training. 2 months. 3 × 120 min. per week	Semi-structured focus interview. Europe ASI	Psychosocial	Yes	Yes	Yes	Qualitative semi-structured focus interviews
Sari et al. [28]	Denmark	RCT	*N* = 117	Outpatient	Running or brisk walking (compared to TAU). 6 months, 2 × 60 min. per week	EQ-5D, ASI	Psychosocial	Yes	No	Yes	No
Welford et al. [31]	Sweden	RCT	*N* = 140	Not seeking treatment	Cardio group training, such as cycling, boxing, dance, aerobics (compared to yoga group training, such as Ashtanga, Hatha, and Yin yoga, and Body Balance or no training). Min. 3 × 60 min. per week	HADS	Psychological	Yes	No	No	No
Xu et al. [39]	China	RCT	*N* = 60	Inpatient	Cycling, running, bodyweight training, stretching (compared to TAU). 3 months, 5 × 60 min. per week	QOL-DA, SDS, SAS	Psychosocial	Yes	No	Yes	No
Zhu et al. [40]	China	Quasi, pilot	*N* = 59	Inpatient	Tai Chi (compared to TAU). 3 months, 5 × 50 min. per week	QOL-DA	Psychosocial	Yes	No	Yes	No
Zhu et al. [41]	China	RCT	*N* = 100	Inpatient	Mind-Body training (compared to TAU, which consisted of Guang Bo led by an instructor for the same duration). 6 months. 3 × 10 min. per day	QOL-DA	Psychosocial	Yes	No	Yes	No
Zhu et al. [42]	China	RCT	*N* = 83	Inpatient	Cardio training with music (compared to TAU). 3 months, 5 × 30 min. per week	HAMA, BDI-II	Psychological	Yes	No	Yes	No

Note. RCT, Randomized Controlled Trial. IG, Intervention Group. TAU, Treatment As Usual. MKG, Matching Control Group. QOL-DA, Quality of Life Scale for drug addiction. HAMA, Hamilton Anxiety Scale. BDI-II, Beck Depression Inventory. SF-36, Short form Health Survey. SDS, Self-rating depression scale. SAS, Self-rating anxiety scale. BSCS, Brief Self-Control Scale. HADS, Hospital anxiety and depression scale. FMI-15, Freiberg Mindfulness Inventory. HRQOLDA, Health Related Quality of Life for Drug Abusers. WHOQOL-BREF, World Health Organization Quality of Life Brief. HSCL-25, Hopkins Symptoms Checklist. EQ-5D, EuroQOL five dimensions questionnaire. ASI, Addiction Severity Index. PSS, Perceived Stress Scale. POMS-ASF, Profile of Mood States-Adapted Short Form. HAM-A, Hamilton anxiety scale. HAM-D, Hamilton depression scale. TMQ, Treatment motivation scale. MAAS, Mindful awareness scale

The group-based physical activity interventions consisted of various combinations of aerobic, strength, and flexibility training. Intervention duration ranged from three weeks to twelve months, with ten studies implementing three-month interventions. Frequency varied from six sessions per week lasting 90 min each to three daily sessions lasting 10 min. In nine studies, the interventions were delivered two to three times per week for 30 to 120 min per session.

The studies used a range of measurement methods. Ten reported that group-based physical activity had positive effects on psychosocial variables, while six found positive effects on psychological variables specifically. One study reported no positive effects on either psychological or social variables, and another study reported improved quality of life without specifying whether the improvement was attributable to psychological or social factors.

The characteristics of the included studies and the findings relevant to the objectives of this scoping review are summarized in Table 1.

### Results related to the primary objectives

The literature search and screening process resulted in 18 studies that examined the outcomes of group-based physical activity on psychosocial variables among individuals with a SUDs. Of these, 15 studies discussed psychosocial factors related to organization, guidance, and interpersonal dynamics that appeared to enhance the reinforcing value of group-based physical activity.

#### Organization

Across studies, several intervention characteristics were consistently associated with increased engagement, adherence, and positive psychosocial outcomes in group-based physical activity among individuals with substance use disorders. A recurring theme was the importance of professionally organized, target-group–adapted settings that provided a clear structure and a predictable, supportive, and psychologically safe environment [27, 29, 30]. Such environments were frequently described as facilitating participation by fostering accountability, peer connection, and a sense of security, conditions considered supportive of recovery-oriented engagement.

Programs that were adapted to participants’ psychological characteristics and physical abilities also appeared more likely to promote adherence and positive experiences. Several studies emphasized that activities should be easy to learn, allow for gradual progression, and accommodate variation in duration and intensity [38, 40]. Conversely, insufficient individualization was associated with reduced motivation and lower adherence [28]. Opportunities for active involvement and individual choice, such as providing input on activities or pacing, were repeatedly highlighted as enhancing engagement and perceived autonomy [26, 38].

The social dimension of group-based physical activity was another central factor across studies. Opportunities for interaction, cooperation, and peer learning were frequently described as increasing motivation and sustaining participation [37, 40]. Several studies noted that supportive and stigma-free group environments facilitated positive and potentially therapeutic relationships, contributing to group cohesion and a sense of belonging [30, 31]. In some interventions, activities were explicitly designed to strengthen the social component of participation, for example through cooperative exercises, outdoor sessions, or group excursions aimed at team building [33, 34, 41].

Enjoyment and experiential differentiation from formal treatment contexts also emerged as salient factors. In one study, participants described group-based physical activity as lighter, voluntary, and more enjoyable than other treatment components, which appeared to facilitate sustained motivation and engagement both in the activity itself and in the broader treatment process [16]. Relatedly, the inclusion of varied and engaging activities, such as rhythmic or aerobic components, was suggested to mitigate boredom and support continued participation [39, 42].

Taken together, these findings suggest that the reinforcing value of group-based physical activity is shaped less by any single intervention element than by the interaction of structured delivery, individual adaptation, and supportive group processes. Rather than operating solely through physical exertion, group-based physical activity appears to function as a socially embedded, experience-based context that may support psychosocial functioning and engagement in recovery-oriented behavior.

#### Guidance

Across several studies, the role of instructors and mentors emerged as a salient psychosocial factor shaping engagement, motivation, and the reinforcing value of group-based physical activity. Interventions that emphasized consistent instructor presence, supportive guidance, and ongoing feedback were commonly described as fostering trust, accountability, and sustained participation.

In some programs, instructors were individuals in recovery who had completed training in exercise instruction and served as relatable role models and sources of social support [30]. Participants described these instructors as embodying attainable recovery-consistent identities, which appeared to enhance motivation and engagement. Relatedly, regular contact with professional teams providing continuous support and feedback was associated with increased adherence, self-esteem, and recognition of individual progress [37, 38].

Several studies highlighted mentoring elements, such as encouragement, guidance, and individualized attention, as key contributors to a supportive group climate. Participants reported that instructor qualities, including attentiveness, consistency, and responsiveness, played an important role in creating an environment in which participants felt supported by both instructors and peers [16, 29]. Instructor-mediated reinforcement, including verbal encouragement, acknowledgment of effort, and recognition of achievements, was repeatedly described as enhancing motivation and perceived value of participation [38].

Instructor continuity also appeared to matter. In one study, having the same instructor throughout the intervention contributed to the instructor becoming an important and trusted social partner, reinforcing participants’ commitment to the activity and the group experience [27]. Taken together, these findings suggest that instructors functioned not merely as facilitators of physical activity, but as central agents in shaping the social and motivational context in which group-based physical activity occurred.

#### Interpersonal factors

Across several studies, social support emerged as a central psychosocial factor that appeared to enhance participation, coping, and engagement in group-based physical activity. Participants frequently described the group context as providing emotional support, opportunities for communication, and reinforcement through social interaction, all of which were perceived as beneficial for mental health and sustained involvement.

Multiple studies highlighted the role of communication and shared activity in fostering psychosocial benefits. For example, group-based aerobic training was described as creating repeated opportunities for interaction, which were associated with improved self-efficacy and mental health outcomes [40, 42]. Similarly, social contact within the group was reported to reinforce continued participation, suggesting that social reinforcement may be an important mechanism supporting adherence [38].

A recurring theme across studies was the importance of belonging, recognition, and relationship building. Group-based physical activity was described as facilitating collaboration, mutual motivation, and a shared commitment to change substance use behavior [27]. At follow-up, participants valued the social relationships and recognition associated with group participation, underscoring the enduring relevance of the social dimension of the intervention. Studies likewise identified group belonging and perceived social support as key factors influencing participation and engagement [29].

Several studies emphasized the supportive function of the group during treatment. In particular, participants described the group as a source of critical support that helped them cope with treatment demands and develop trust in others [16, 30] similarly focused on the development of social and human capital through group-based physical activity, suggesting that these relational resources may contribute to broader recovery-relevant outcomes. In contrast, insufficient social support was proposed as a potential explanation for declining motivation and adherence in one study [28], further underscoring the salience of social relationships in this context.

Notably, no information regarding psychosocial factors that appeared to enhance the reinforcing value of group-based physical activity was reported in the studies by Gaihre et al., [32], Huang et al., [35], or Kiraz and Yıldırım [36]. This absence highlights variability in the extent to which psychosocial processes were explicitly examined or reported across the included literature.

### Results related to the secondary objectives

Of the 18 included studies, four emphasized interaction, communication, cooperation, and cohesion within their interventions. In Fitzgerald et al. [30], one of the aims was to investigate how group-based physical activity could promote social and human capital. The intervention took place in a fitness center and explicitly relied on community-based approaches and interaction with peers in recovery. Giménez-Meseguer et al. [34] incorporated engaging activities designed to strengthen the social component and support motivation and participation. Roessler, [27] emphasized enhancing participant interaction during training sessions with the goal of fostering mutual motivation for behavioral change. In Nowakowski-Sims and Bullard [29], the primary aim was to examine the role of group-based physical activity in supporting recovery and maintaining abstinence; the authors noted that group-based activity can generate intrinsic motivation to continue exercising and that social variables were central to participants’ wellbeing.

In 17 of the studies included, group-based physical activity was provided in addition to other psychosocial treatments. In one of these studies [29], some participants were not engaged in concurrent psychosocial treatment. One study included only participants who were not seeking treatment [31].

## Discussion

### Discussion of the primary objectives

This scoping review identified 18 studies published between 2010 and 2025 that examined the outcomes of group-based physical activity on psychosocial variables among individuals with a SUDs. Fifteen of these studies described psychosocial factors that appeared to enhance the reinforcing value of group-based physical activity. These factors included the group setting itself, a safe and inclusive environment, variation in activities, supportive and trained instructors, opportunities for individualization and autonomy, recognition and mastery experiences, and social support and belonging. Many of these factors align with those identified by Vella et al. [17] as optimizing the mental-health benefits of physical activity.

The influence of motivating operations on reinforcers, however, is variable and depends on factors such as emotional states and individual preferences for particular stimuli or activities [43]. For example, high anxiety may function as an abolishing operation that reduces the reinforcing value of group-based physical activity, whereas low anxiety may function as an establishing operation that increases its reinforcing value. Similarly, social support and perceived mastery may serve as establishing operations that enhance reinforcement, while the absence of social support or a lack of mastery experiences may act as abolishing operations. Preferences for specific group-based activities also vary: football, for instance, may function as an establishing operation for an individual who enjoys football but as an abolishing operation for someone who prefers golf.

Addiction is characterized by impulsive decision-making [44], which may affect choices between group-based physical activity and more immediate reinforcers such as substances. However, contingency-specifying stimuli may function as establishing operations, particularly when consequences are delayed, if autonomy and social support are important to the individual. These stimuli include information conveying that a group-based activity emphasizes autonomy, support, and belonging. Personal rules, such as “When I go to training, I feel competent and I feel better afterward. I also appreciate the support and positive social relationships,” may function both as coping strategies and as verbal stimuli that influence the reinforcing value of group-based physical activity. According to Michael (2007), such rules may operate as reflexive conditioned motivating operations [45] signaling that improvement is forthcoming, and as contingency-specifying function-altering stimuli [46].

Thus, the effects of psychosocial factors on group-based physical activity cannot be unambiguously classified as either establishing or abolishing operations. In fact, an important consideration when interpreting our findings is that most group‑based physical activity interventions were delivered in conjunction with other psychosocial treatments, making it difficult to isolate the specific contribution of the group format or the physical activity component alone. Rather than functioning as a stand‑alone intervention, physical activity was often embedded within broader treatment programs, where group processes, therapeutic content, and structured exercise may interact in synergy. In this context, psychosocial outcomes may reflect the combined influence of physical engagement, social interaction, and concurrent therapeutic support, rather than a single operation.

Accordingly, our findings should be understood as illustrating how group‑based physical activity operates within diverse treatment environments, highlighting potential mechanisms and contextual features that may enhance psychosocial functioning, rather than delineating discrete intervention effects.

### Discussion of the secondary objectives

Although social support and the benefits derived from social activity are central to the prevention and treatment of addiction [47], only four of the included studies emphasized interaction, communication, cooperation, and cohesion in their interventions. These factors are associated with positive therapeutic outcomes through collaborative and problem-solving activities [19]. This suggests a limited focus on the group component within the included interventions, despite evidence that group-based physical activity may enhance quality of life to a greater extent than individual exercise [18].

Furthermore, group-based physical activity was typically delivered alongside other forms of treatment, such as individual or group therapy, as well as additional potentially confounding factors, including support and motivation provided by staff and other service users. Among the nine randomized controlled trials included in this review where control groups received comparable treatment within the same clinical setting, all but one [26] demonstrated positive effects of group-based physical activity on psychosocial variables. This suggests that the physical activity itself contributed to changes in psychosocial outcomes. However, these findings do not necessarily clarify the contribution of the group component, as the observed effects may be attributable to physical activity alone rather than to social interaction or group-based processes.

### Limitations

No previous reviews were identified that examined group-based physical activity in relation to psychosocial outcomes among individuals with SUDs. A scoping review was therefore deemed appropriate to map the breadth of existing research and to identify key features and gaps within this emerging literature. Unlike systematic reviews, scoping reviews are less constrained by predefined criteria regarding interventions, control conditions, and outcome variables, allowing for greater flexibility when addressing complex or multifaceted topics. Consistent with this methodology, the aim of the present review was to describe and synthesize existing evidence rather than to conduct a formal quality appraisal of individual studies.

All included studies were peer-reviewed, suggesting a baseline level of methodological rigor; however, risk of bias was not formally assessed, in line with established guidance for scoping reviews. While a formal appraisal could have provided a more nuanced evaluation of the internal validity of individual findings, its absence reflects the review’s descriptive and mapping-oriented purpose rather than a claim regarding intervention effectiveness.

The exclusion criteria applied in this review also introduce systematic sources of bias that should be considered when interpreting the findings. Restricting inclusion to peer-reviewed studies may have contributed to publication bias, potentially favoring well-resourced research contexts and studies reporting clearer or more favorable outcomes. In addition, the exclusion of studies conducted in certain settings or using less standardized outcome measures may have resulted in the underrepresentation of more complex or resource-constrained clinical environments in which group-based physical activity is implemented. As a result, the patterns identified in this review are best interpreted as reflecting how such interventions function under relatively structured and well-documented conditions rather than as comprehensive evidence applicable across all SUD treatment contexts. Furthermore, study screening, data charting, and synthesis were conducted by a single reviewer (i.e., the first author), which may have increased the risk of selection and interpretation bias.

Although several included studies employed randomized controlled trials (RCTs) designs, data were also drawn from quasi-experimental and qualitative studies, contributing to heterogeneity in methodological approaches. In synthesizing the literature, our review did not systematically differentiate findings according to study design, and RCTs were therefore not afforded distinct analytic prominence relative to non-randomized studies. Consequently, evidence derived from qualitative and quasi-experimental designs, primarily illuminating contextual, experiential, and implementation-related factors, received the same narrative attention to findings from RCTs. This may have reduced the clarity with which design-specific inferential strength is communicated, particularly with respect to claims regarding psychosocial effects.

Because group-based physical activity was frequently delivered alongside other psychosocial treatments, the independent contribution of the group or physical activity components cannot be disentangled, and observed psychosocial outcomes likely reflect interactive or additive effects. Additionally, substantial heterogeneity across studies in intervention type, duration, intensity, delivery, and treatment context limits direct comparability and necessitates interpreting the findings as indicative of recurring psychosocial themes rather than intervention-specific effects.

Taken together, these limitations do not undermine the descriptive value of the review but do delimit the scope of inference: our findings should be understood as informing theory-consistent applications and guiding future research priorities rather than as providing definitive conclusions regarding the effectiveness of group-based physical activity for individuals with SUDs.

## Conclusion

This scoping review identified multiple studies examining the outcomes of group-based physical activity on psychosocial variables among individuals with SUDs. The findings suggest that psychosocial factors such as inclusive environment, professional guidance, perceived autonomy, belonging, and social support may enhance the reinforcing value of group-based physical activity.

The review also revealed a notable knowledge gap regarding the group component of these interventions and its influence on psychosocial outcomes. Few studies explicitly emphasized interaction, communication, cooperation, and cohesion, despite the potential relevance of these elements for strengthening social processes and promoting recovery.

Based on the findings of this review, future research should investigate group-based physical activity interventions that deliberately incorporate interaction, communication, cooperation, and cohesion, and examine how these components affect psychosocial variables among individuals with SUDs. Such research should consider group-based activity as an adjunct to psychosocial treatment, as a bridging intervention between inpatient treatment and no treatment, and as a standalone intervention. Further research is also warranted to identify psychosocial factors that generally increase the reinforcing value of group-based physical activity and function as establishing operations for motivation.

Providing a broad range of accessible, substance-free group-based physical activities grounded in psychosocial factors that may function as motivating operations, and thus potentially enhance the reinforcing value of participation, may represent a positive and cost-effective approach to preventing and reducing substance-related challenges at both individual and societal levels. Moreover, cultural selection processes may help sustain such interventions: reciprocal influence among participants can strengthen desired behavioral patterns and foster a shared supportive environment, creating enduring effects that maintain and reinforce participation in group-based physical activity.

## Supplementary Information

Below is the link to the electronic supplementary material.

Supplementary Material 1

## Data Availability

All data analyzed in this study are from publicly available sources cited in the manuscript.

## References

[1] Bramness JG. Rusmiddellidelser i Norge. In: Folkehelserapporten – Helsetilstanden i Norge [Public Health Report: Substance Abuse Disorders in Norway]. Oslo: Folkehelseinstituttet. 2022. https://www.fhi.no/he/folkehelserapporten/psykisk-helse/rusmiddellidelser/.

[2] Whiteford HAP, Degenhardt LP, Rehm JP, Baxter AJMPH, Ferrari AJB, Erskine HEB, Charlson FJMPH, Norman REP, Flaxman ADP, Johns NBA, Burstein RBA, Murray CJLP, Vos TP. Global burden of disease attributable to mental and SUDs: Findings from the Global Burden of Disease Study 2010. Lancet. 2013;382(9904):1575–86. 10.1016/S0140-6736(13)61611-6. 23993280 10.1016/S0140-6736(13)61611-6

[3] Lappan SN, Brown AW, Hendricks PS. Dropout rates of in-person psychosocial SUDs treatments: A systematic review and meta‐analysis. Addiction. 2020;115(2):201–17. 10.1111/add.14793. 31454123 10.1111/add.14793

[4] United Nations Association of Norway. Mål 3: God helse og livskvalitet [Goal 3: Good health and quality of life] [Internet]. 2025. https://fn.no/om-fn/fns-baerekraftsmaal/god-helse-og-livskvalitet

[5] Norwegian Government (2022-2023). Meld. St. 23 Opptrappingsplan for psykisk helse (2023–2033) [Mental health escalation plan ]. Helse- og omsorgsdepartementet. 2023. https://www.regjeringen.no/no/dokumenter/meld.-st.-23-20222023/id2983623/.

[6] World Health Organization. Comprehensive mental health action plan 2013–2030. World Health Organization. 2021. https://iris.who.int/handle/10665/345579.

[7] United Nations Office on Drugs and Crime & World Health Organization. International standards for the treatment of drug use disorders: Revised edition incorporating results of field-testing. UNODC. 2020. https://www.unodc.org/documents/drug-prevention-and-treatment/UNODC-WHO_International_Standards_Treatment_Drug_Use_Disorders_April_2020.pdf.

[8] Malerbakken A, Malmberg CM, Walderhaug E. Ut av rusbehanding, inn i ensomhet? [Out of drug treatment, into loneliness?] Magasinet Psykisk Helse. 2022. https://psykiskhelse.no/magasinet/ut-av-rusbehandling-inn-i-ensomhet/.

[9] McGaffin BJ, Deane FP, Kelly PJ, Blackman RJ. Social support and mental health during recovery from drug and alcohol problems. Addict Res Theory. 2018;26(5):386–95. 10.1080/16066359.2017.1421178.

[10] Jia D, Zhang K, Xu Y. the relationship between social support and relapse tendency among those who struggle with drug addiction: Multiple mediators of exercise self-efficacy and health-related quality of life. J Drug Issues. 2024;54(1):120–33. 10.1177/00220426231152912.

[11] Prins EH. Maturing out and the dynamics of the biographical trajectories of hard drug addicts. Forum: Qualitative Social Res. 2008;9(1). https://www.qualitative-research.net/index.php/fqs/article/view/333.

[12] Tucker JA, Vuchinich RE, Rippens PD. Predicting natural resolution of alcohol-related problems: A prospective behavioral economic analysis. Experimental Clin Psychopharmacol. 2002;10(3):248–57. 10.1037/1064-1297.10.3.248. 10.1037//1064-1297.10.3.248 12233985

[13] Nystad W, Ekelund U. Folkehelserapporten: Fysisk aktivitet i Norge [Public Health Report: Physical activity in Norway]. Folkehelseinstituttet. 2023. https://www.fhi.no/he/fr/folkehelserapporten/levevaner/fysisk-aktivitet/.

[14] Zschucke E, Heinz A, Ströhle A. Exercise and physical activity in the therapy of SUDs. Sci World J. 2012;2012(1):1–19. 10.1100/2012/901741. 10.1100/2012/901741 PMC3354725 22629222

[15] Piche F, Daneau C, Plourde C, Girard S, Romain AJ. Characteristics and impact of physical activity interventions during SUDs treatment excluding tobacco: A systematic review. PLoS ONE. 2023;18(4):1–16. 10.1371/journal.pone.0283861. 10.1371/journal.pone.0283861 PMC10132651 37099488

[16] Piche F, Girard S, Plourde C, Romain AJ. Physical activity during a treatment for SUDs: A qualitative study. Ment Health Phys Act. 2024;26:1–9. 10.1016/j.mhpa.2024.100590.

[17] Vella SA, Aidman E, Teychenne M, Smith JJ, Swann C, Rosenbaum S, White RL, Lubans DR. Optimising the effects of physical activity on mental health and wellbeing: A joint consensus statement from Sports Medicine Australia and the Australian Psychological Society. J Sci Med Sport. 2023;26(2):132–9. 10.1016/j.jsams.2023.01.001. 36737260 10.1016/j.jsams.2023.01.001

[18] Gillison FB, Skevington SM, Sato A, Standage M, Evangelidou S. The effects of exercise interventions on quality of life in clinical and healthy populations: A meta-analysis. Soc Sci Med. 2009;68(9):1700–10. 10.1016/j.socscimed.2009.02.028. 19297065 10.1016/j.socscimed.2009.02.028

[19] Thal SB, Maunz LA, Quested E, Bright SJ, Myers B, Ntoumanis N. Behavior change techniques in physical activity interventions for adults with SUDs: A systematic review. Psychol Addict Behav. 2023;37(3):416–33. 10.1037/adb0000842. 35666890 10.1037/adb0000842

[20] Thomas K, Nilsson E, Festin K, Henriksson P, Lowén M, Löf M, Kristenson M. Associations of psychosocial factors with multiple health behaviors: a population-based study of middle-aged men and women. Int J Environ Res Public Health. 2020;17(4):1239. 10.3390/ijerph17041239. 32075162 10.3390/ijerph17041239 PMC7068361

[21] The Norwegian Directorate of Health. 1.2. Begrepsbruk [1.2. Terminology]. The Norwegian Directorate of Health; 2016 Mar 17 [cited 2025 Oct 2]. https://www.helsedirektoratet.no/veiledere/psykososiale-tiltak-ved-kriser-ulykker-og-katastrofer/innledning-og-begrepsbruk/begrepsbruk

[22] Tricco AC, Lillie E, Zarin W, O’Brien KK, Colquhoun H, Levac D, Moher D, Peters MDJ, Horsley T, Weeks L, Hempel S, Akl EA, Chang C, McGowan J, Stewart L, Hartling L, Aldcroft A, Wilson MG, Garritty C, Straus SE. PRISMA extension for scoping reviews (PRISMA-ScR): Checklist and explanation. Ann Intern Med. 2018;169(7):467–73. 10.7326/M18-0850. 30178033 10.7326/M18-0850

[23] Smith SA, Duncan AA. Systematic and Scoping Reviews: A Comparison and Overview. Semin Vasc Surg. 2022;35(4):464–9. 10.1053/j.semvascsurg.2022.09.001. 36414363 10.1053/j.semvascsurg.2022.09.001

[24] Pollock D, Evans C, Menghao Jia R, Alexander L, Pieper D, de Brandão É, Peters MDJ, Tricco AC, Khalil H, Godfrey CM, Saran A, Campbell F, Munn Z. How-to: scoping review? J Clin Epidemiol. 2024;176:111572–111572. 10.1016/j.jclinepi.2024.111572. 39426499 10.1016/j.jclinepi.2024.111572

[25] McHugh ML. Interrater reliability: The kappa statistic. Biochemia Med. 2012;22(3):276–82. 10.11613/bm.2012.031. PMC3900052 23092060

[26] Colledge F, Vogel M, Dursteler-Macfarland K, Strom J, Schoen S, Puhse U, Gerber M. A pilot randomized trial of exercise as adjunct therapy in a heroin-assisted treatment setting. J Subst Abuse Treat. 2017;76:49–57. 10.1016/j.jsat.2017.01.012. 28143679 10.1016/j.jsat.2017.01.012

[27] Roessler KK. Exercise treatment for drug abuse: A Danish pilot study. Scand J Public Health. 2010;38(6):664–9. 10.1177/1403494810371249. 20529968 10.1177/1403494810371249

[28] Sari S, Bilberg R, Sogaard Nielsen A, Roessler KK. The effect of exercise as adjunctive treatment on quality of life for individuals with alcohol use disorders: A randomized controlled trial. BMC Public Health. 2019;19(1):1–8. 10.1186/s12889-019-7083-8. 31185955 10.1186/s12889-019-7083-8 PMC6558793

[29] Nowakowski-Sims E, Bullard K. Relearning to live life without substances: A grounded theory of the impact of group physical exercise on sobriety. J Social Work Pract Addictions. 2018;18(3):305–24. 10.1080/1533256X.2018.1485575.

[30] Fitzgerald C, Webb C, McNally C. Lift Yourself Up: The short-term associations between strength training and mood states and the longer term development of physical capital and grit among people recovering from SUDs. Health Promot Pract. 2024;25(5):845–54. 10.1177/15248399241245051. 38686654 10.1177/15248399241245051

[31] Welford P, Gunillasdotter V, Andreasson S, Hallgren M. Effects of physical activity on symptoms of depression and anxiety in adults with alcohol use disorder (FitForChange): Secondary outcomes of a randomised controlled trial. Drug Alcohol Depend. 2022;239:1–9. 10.1016/j.drugalcdep.2022.109601. 10.1016/j.drugalcdep.2022.109601 35994841

[32] Gaihre A, Sasidharan RK, Bista S, Poudel L, Khadka R, Rajbhandari B. Impact of yoga and physical exercise on psychological wellbeing among substance abusers: A randomized controlled trial. J Complement Integr Med. 2020;20(1):241–9. 10.1515/jcim-2020-0506. 10.1515/jcim-2020-0506 34506694

[33] Giesen ES, Zimmer P, Bloch W. Effects of an exercise program on physical activity level and quality of life in patients with severe alcohol dependence. Alcoholism Treat Q. 2016;34(1):63–78. 10.1080/07347324.2016.1113109.

[34] Giménez-Meseguer J, Tortosa-Martínez J, Remedios Fernández-Valenciano MDL. Benefits of exercise for the quality of life of drug-dependent patients. J Psychoactive Drugs. 2015;47(5):409–16. 10.1080/02791072.2015.1102991. 26595433 10.1080/02791072.2015.1102991

[35] Huang X, Wang X, Shao Y, Lin A, Zhang Z, Qi H, Sun C, Yang H. Effects of health qigong exercise on sleep and life quality in patients with drug abuse. Hong Kong J Occup Therapy. 2023;36(1):13–9. 10.1177/15691861231156002. 10.1177/15691861231156002 PMC10273793 37332297

[36] Kiraz S, Yıldırım S. The effect of regular exercise on depression, anxiety, treatment motivation and mindfulness in addiction: A randomized controlled trial. J Subst Use. 2023;28(4):643–50. 10.1080/14659891.2023.2194417.

[37] Monton-Martinez R, Ballester-Ferrer JA, Baladzhaeva S, Sempere-Ruiz N, Casanova-Lizon A, Roldan A, Pastor D, Sarabia JM, Javaloyes A, Pena-Gonzalez I, Moya-Ramon M. exploring the impact of web-based vs. in-person exercise training on benefits and adherence in SUDs interventions: A pilot study. Healthcare. 2024;12(6):1–12. 10.3390/healthcare12060684. 10.3390/healthcare12060684 PMC10969899 38540648

[38] Muller AE, Clausen T. Group exercise to improve quality of life among SUDs patients. Scand J Public Health. 2015;43(2):146–52. 10.1177/1403494814561819. 25527637 10.1177/1403494814561819

[39] Xu J, Zhu Z, Liang X, Huang Q, Zheng T, Li X. Effects of moderate-intensity exercise on social health and physical and mental health of methamphetamine-dependent individuals: A randomized controlled trial. Front Psychiatry Front Res Foundation. 2022;13:1–11. 10.3389/fpsyt.2022.997960. 10.3389/fpsyt.2022.997960 PMC9539410 36213929

[40] Zhu D, Xu D, Dai G, Wang F, Xu X, Zhou D. Beneficial effects of Tai Chi for amphetamine-type stimulant dependence: A pilot study. Am J Drug Alcohol Abus. 2016;42(4):469–78. 10.3109/00952990.2016.1153646. 10.3109/00952990.2016.1153646 27211290

[41] Zhu D, Jiang M, Xu D, Schollhorn WI. Long-term effects of mind-body exercises on the physical fitness and quality of life of individuals with SUDs: A randomized trial. Front Psychiatry. 2020;11:1–11. 10.3389/fpsyt.2020.528373. 33391039 10.3389/fpsyt.2020.528373 PMC7775308

[42] Zhu T, Tao W, Peng B, Su R, Wang D, Hu C, Chang YK. Effects of a group-based aerobic exercise program on the cognitive functions and emotions of SUDs patients: a randomized controlled trial. Int J Mental Health Addict. 2021;20(4):2349–65. 10.1007/s11469-021-00518-x.

[43] Poling A, Lotfizadeh A, Edwards TL. Predicting reinforcement: utility of the motivating operations concept. Behav Analyst. 2017;40(1):49–56. 10.1007/s40614-017-0091-z. 10.1007/s40614-017-0091-z PMC6701243 31976977

[44] Locey ML, Buddiga NR, Nomicos B, L., Smith CA. Commodity discounting: Obstacles and solutions. Psychol Addict Behav. 2023;37(1):25–36. 10.1037/adb0000879. 36048066 10.1037/adb0000879

[45] Langthorne P, McGill P. A tutorial on the concept of the motivating operation and its importance to application. Behav Anal Pract. 2009;2(2):22–31. 10.1007/BF03391745. 22477704 10.1007/BF03391745 PMC2859803

[46] Schlinger H, Blakely E. Function-altering effects of contingency-specifying stimuli. Behav Analyst. 1987;10(1):41–5. 10.1007/BF03392405. 10.1007/BF03392405 PMC2741931 22477959

[47] Rachlin H. The lonely addict. The Science of Self-Control. Harward University. University Press; 2000:82–107.

