# Supplementary material for: Psychosocial outcomes of group-based physical activity among individuals with substance use disorders: a scoping review
Source: Addict Sci Clin Pract. 2026 Jun 16;21:54. doi: 10.1186/s13722-026-00685-z (PMC13330098; doi:10.1186/s13722-026-00685-z)
Supplement: Supplementary file 1 — Supplementary Material 1 [file 13722_2026_685_MOESM1_ESM.docx]

**Appendix A**

*Databases and references used in the search*

| Database | Platform | Year | Description |
| --- | --- | --- | --- |
| MEDLINE | Ovid | 2010 til 21. januar 2025 | Bibliographic database with information and summaries of biomedical literature. Published by the U.S. National Library of Medicine (NLM) |
| PsychINFO | Ovid | 2010 til 21. januar 2025 | Bibliographic database of journal articles, books, book chapters and dissertations relevant to psychology and related fields. Published by the American Psychological Association (APA) |
| Scopus | https://www.scopus.com | 2010 til 21. januar 2025 | Bibliographic database with access to academic journals and other academic material. Published by Elsevier |
| Cochrane Library | Ovid | 2010 til 21. januar 2025 | Bibliographic database with access to systematic reviews and controlled, randomized trials. Published by Cochrane Library in collaboration with U.S.NLM, Elsevier and others |

**Appendix B**

*Overview of the final search in databases 21 January 2025*

|  | | | | |
| --- | --- | --- | --- | --- |
| Database | Physical group activity | Drug addiction | Results | Filter |
| Cochrane Library |  |  |  |  |
|  | (group* NEXT (exercis* OR activ* OR physical OR training)) OR ((activ* OR exercis* OR training OR aerobic) NEXT (group* OR "group-based")) or "street soccer" or soccer or "workout classes" or "walking groups" or "workout class" or "walking group" or volleyball or basketball or rugby or hockey or "group aerobic" or "group aerobics" or "pilates class" or "yoga class" or Zumba or "spin class" or "dance class" "pilates classes" or "yoga classes" or Zumba or "spin classes" or "dance classes" or dodgeball or football | substance use disorder* or alcohol use disorder or substance addict* or substance use* or substance abuse or drug addict* or drug abuse or alcohol abuse or drug dependen* or alcohol dependen* or substance dependen* or stimulant dependen* or alcohol drink* or alcohol rehabilitation program* or narcotic* or morphine or opiate or opioid or heroin or cocaine or methamphetamine or amphetamine or crack or cannabis or marijuana or subutex or methadone or buvidal or buprenorphine or polysubstance abuse | 1085 | Search in title, abstract and keywords. Publication year from January 2010 to January 2025, in *Trials.* Variations of words have been searcched. |
| Scopus |  |  |  |  |
|  | ("group exercis*" or "group activ*" or "group-based exercis*" or "group-based activ*" or "group-based physical activ*" or "physical activ* group intervention" or "group-based aerobic exercis*" or "exercis* group*" or "group training" or "group-based aerobic exercis*" or "group physical exercis*" or "street soccer" or soccer or "workout class*" or "walking group*" or volleyball or basketball or rugby or hockey or "group aerobic*" or "pilates class*" or "yoga class*" or Zumba or "spin class*" or "dance class*" or dodgeball or football) AND | ("substance use* disorder*" or "alcohol use* disorder*" or "substance addict*" or "substance use*" or "substanc* abuse" or "drug addict*" or "drug abuse" or "alcohol abuse" or "drug dependen*" or "alcohol dependen*" or "substanc* dependen*" or "stimulant dependen*" or "drug dependen*" or "alcohol drink*" or "alcohol rehabilitation program*" or narcotic* or morphine or opiate or opioid or heroin or cocaine or methamphetamine or amphetamine or crack or cannabis or marijuana or subutex or methadone or buvidal or buprenorphine or "polysubstance abuse") | 636 | Search in title, abstract, abstract and keywords. Publication years from 2009 to 2025. Document type restricted to article and review. Language limited to English |
| MEDLINE |  | |  |  |
| Search string |  | | Results | Type |
| 1 | substance-related disorders/ or alcoholism/ or drug misuse/ or prescription drug misuse/ or marijuana abuse/ or heroin dependence/ or morphine dependence/ or opium dependence/ or substance abuse, intravenous/ or substance abuse, oral/ | | 214105 | Advanced |
| 2 | ("substance use* disorder*" or "alcohol use* disorder*" or "substance addict*" or "substance use*" or "substanc* abuse" or "drug addict*" or "drug abuse" or "alcohol abuse" or "drug dependen*" or "alcohol dependen*" or "substanc* dependen*" or "stimulant dependen*" or "drug dependen*" or "alcohol drink*" or "alcohol rehabilitation program*" or narcotic* or morphine or opiate or opioid or heroin or cocaine or methamphetamine or amphetamine or crack or cannabis or marijuana or subutex or methadone or buvidal or buprenorphine or "polysubstance abuse").mp. | | 564508 | Advanced |
| 3 | 1 or 2 | | 641883 | Advanced |
| 4 | ("group exercis*" or "group activ*" or "group-based exercis*" or "group-based activ*" or "group-based physical activ*" or "physical activ* group intervention" or "group-based aerobic exercis*" or "exercis* group*" or "group training" or "group-based aerobic exercis*" or "group physical exercis*" or "street soccer" or soccer or "workout class*" or "walking group*" or volleyball or basketball or rugby or hockey or "group aerobic*" or "pilates class*" or "yoga class*" or Zumba or "spin class*" or "dance class*" or dodgeball or football).mp. | | 57257 | Advanced |
| 5 | 3 and 4 | | 586 | Advanced |
| 6 | Limit 5 to yr=»2010-Current» | | 408 | Advanced |
| 7 | Limit 6 to (english or norwegian) | | 397 | Advanced |
| PsycINFO |  | |  |  |
| 1 | "substance use disorder"/ or exp "alcohol use disorder"/ or "cannabis use disorder"/ or exp drug abuse/ or drug dependency/ or exp "opioid use disorder"/ or exp drug addiction/ | | 143037 | Advanced |
| 2 | ("substance use* disorder*" or "alcohol use* disorder*" or "substance addict*" or "substance use*" or "substanc* abuse" or "drug addict*" or "drug abuse" or "alcohol abuse" or "drug dependen*" or "alcohol dependen*" or "substanc* dependen*" or "stimulant dependen*" or "drug dependen*" or "alcohol drink*" or "alcohol rehabilitation program*" or narcotic* or morphine or opiate or opioid or heroin or cocaine or methamphetamine or amphetamine or crack or cannabis or marijuana or subutex or methadone or buvidal or buprenorphine or "polysubstance abuse").mp. | | 272015 | Advanced |
| 3 | 1 or 2 | | 291888 | Advanced |
| 4 | exercise/ or physical activity/ or aerobic exercise/ | | 55543 | Advanced |
| 5 | ("group exercis*" or "group activ*" or "group-based exercis*" or "group-based activ*" or "group-based physical activ*" or "physical activ* group intervention" or "group-based aerobic exercis*" or "exercis* group*" or "group training" or "group-based aerobic exercis*" or "group physical exercis*" or "street soccer" or soccer or "workout class*" or "walking group*" or volleyball or basketball or rugby or hockey or "group aerobic*" or "pilates class*" or "yoga class*" or Zumba or "spin class*" or "dance class*" or dodgeball or football).mp. | | 21623 | Advanced |
| 6 | 4 or 5 | | 74668 | Advanced |
| 7 | 3 and 6 | | 1812 | Advanced |
| 8 | Limit 7 to (peer reviewed journal and “300 adulthood (age 18 yrs and older)” and (english or Norwegian) and yr=”2010 -Current”) | | 776 | Advanced |
